# Simultaneous Electrochemical Exfoliation and Functionalization of 2H-MoS_2_ for Supercapacitor Electrodes

**DOI:** 10.1021/acsanm.3c03322

**Published:** 2023-10-02

**Authors:** Yuling Zhuo, Ian A. Kinloch, Mark A. Bissett

**Affiliations:** Department of Materials, National Graphene Institute, University of Manchester, Oxford Road, Manchester M13 9PL, United Kingdom

**Keywords:** 2D materials, graphene, molybdenum disulfide, aryl diazonium salts, functionalization, supercapacitors

## Abstract

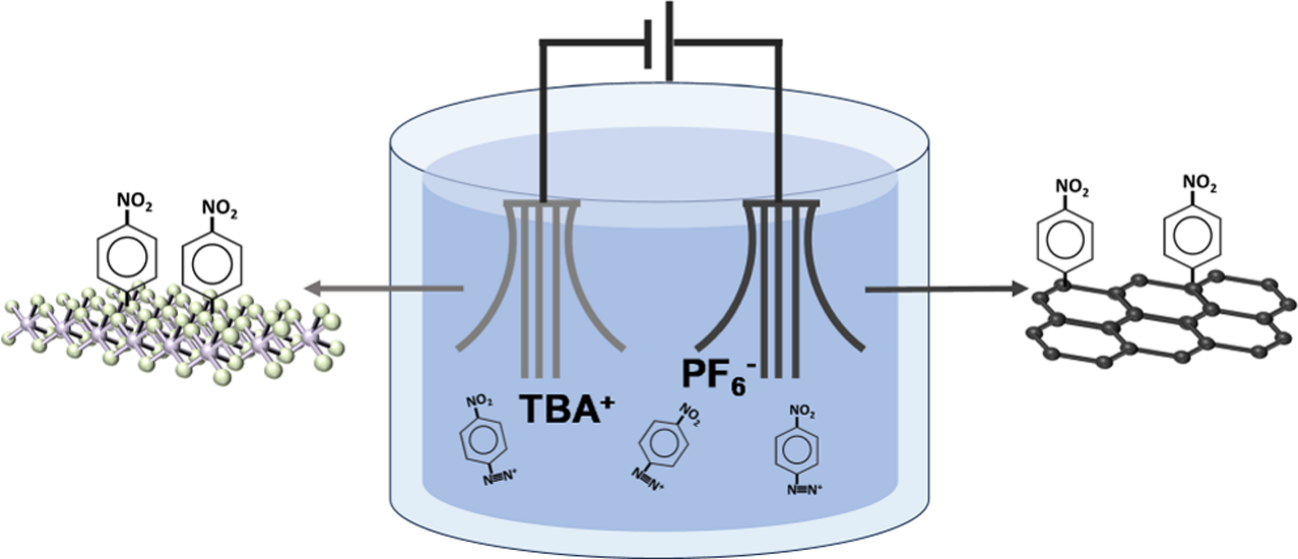

MoS_2_ is a promising semiconducting material that has been widely studied for applications in catalysis and energy storage. The covalent chemical functionalization of MoS_2_ can be used to tune the optoelectronic and chemical properties of MoS_2_ for different applications. However, 2H-MoS_2_ is typically chemically inert and difficult to functionalize directly and thus requires pretreatments such as a phase transition to 1T-MoS_2_ or argon plasma bombardment to introduce reactive defects. Apart from being inefficient and inconvenient, these methods can cause degradation of the desirable properties and introduce unwanted defects. Here, we demonstrate that 2H-MoS_2_ can be simultaneously electrochemically exfoliated and chemically functionalized in a facile and scalable procedure to fabricate functionalized thin (∼4 nm) MoS_2_ layers. The aryl diazonium salts used for functionalization have not only been successfully covalently grafted onto the 2H-MoS_2_, as verified by X-ray photoelectron spectroscopy (XPS) and Raman spectroscopy, but also aid the exfoliation process by increasing the interlayer spacing and preventing restacking. Electrochemical energy storage is one application area to which this material is particularly suited, and characterization of supercapacitor electrodes using this exfoliated and functionalized material revealed that the specific capacitance was increased by ∼25% when functionalized. The methodology demonstrated for the simultaneous production and functionalization of two-dimensional (2D) materials is significant, as it allows for control over the flake morphology with increased repeatability. This electrochemical functionalization technique could also be extended to other types of transition-metal dichalcogenides (TMDs), which are also typically chemically inert with different functional species to adjust to specific applications.

## Introduction

MoS_2_, which is similar to graphene in terms of structure, is a layered transition-metal dichalcogenide (TMD). Compared to some other TMDs, MoS_2_ is naturally abundant and relatively cheap and thus has attracted increasing attention.^1^ It has been widely investigated for applications in electronics, photonics, sensing, energy storage, etc.^2^ Exfoliated MoS_2_ can be obtained through mechanical exfoliation, liquid-phase exfoliation, electrochemical exfoliation, and chemical exfoliation.^3−8^

Aryl diazonium salts, which have a common structure of N_2_^+^-Aryl-R, stand out as surface modifiers due to their simplicity of functionalization and characterization. In addition, –R can be replaced by other types of functional groups according to the desired application. They are therefore widely used to modify the surfaces of materials for sensors, electrodes, catalysts, and other applications.^9,10^ They were first used to functionalize glassy carbon by Pinson et al., which then opened up the prospect of surface modification of other materials using aryl diazonium salts.^11^ Recent reports have shown the importance of functionalizing MoS_2_ in enhancing their properties for hydrogen evolution reaction (HER) and electronic and sensing applications.^12,13^ However, 2H-MoS_2_ is typically inert to chemical functionalization because its Mo atoms are saturated with chalcogen atoms and thus 2H-MoS_2_ is difficult to functionalize directly.^14,15^ As a consequence, recent reports on the functionalization of 2H-MoS_2_ include pretreatment of the flakes prior to functionalization. For example, Benson et al. reported the functionalization of MoS_2_ for applications in HER by first changing the phase of MoS_2_ from 2H to 1T through chemical exfoliation using *n*-butyllithium, followed by a functionalization with aryl diazonium salts.^13^ Although functionalization in this way is successful, it results in losses of desired properties of 2H-MoS_2_ such as the semiconducting electronic structure.^12^ Chu et al. synthesized diazonium-functionalized MoS_2_ by bombarding the MoS_2_ with Ar plasma in order to create S-vacancies, which enhanced the functionalization.^12^ Nevertheless, this may lead to unwanted defects on the basal planes, which could be detrimental to desired properties.

Previously, we have demonstrated how graphene can be simultaneously electrochemically exfoliated and functionalized,^16^ as well as combined with 1T-MoS_2_,^17^ and here we extend this work to 2H-MoS_2_. In order to avoid either losing the desirable semiconducting properties of MoS_2_ or creating unwanted defects, we demonstrate a convenient and efficient way of functionalizing 2H-MoS_2_ directly without any pretreatment. Briefly, thin functionalized MoS_2_ (fct-EEM) flakes of high quality can be obtained electrochemically through simultaneous exfoliation and functionalization using aryl diazonium salts and TBA^+^ (tetrabutylammonium) in the electrolyte. In this simultaneous exfoliation and functionalization process, the MoS_2_ crystal was used as a cathode and graphite tape as the anode; therefore, functionalized graphene can also be obtained at the same time as a byproduct. Unfunctionalized pristine MoS_2_ and graphene were also fabricated as references to the functionalized materials. The functionalized MoS_2_ and functionalized graphene (fct-EEG) were characterized by Raman spectroscopy and X-ray photoelectron spectroscopy (XPS). We show that the functionalization for both 2H-MoS_2_ and the graphene was successful. The existence of the aryl diazonium salts not only functionalizes the MoS_2_ but also assists with the exfoliation process, leading to thinner MoS_2_ flakes. We then performed electrochemical measurements including cyclic voltammetry (CV), galvanostatic charge–discharge (GCD), and electrochemical impedance spectroscopy (EIS) on electrodes made of fct-EEM/fct-EEG composites to see how the functionalization can affect the electrochemical properties compared to unfunctionalized composite electrode. The functionalization increases the contribution of the pseudocapacitance in the electrode, resulting in a higher specific capacitance. This promising technique for the exfoliation of functionalization of MoS_2_ and graphene by aryl diazonium salts opens up prospects in other applications such as sensing, HER, and surface-enhanced Raman scattering (SERS).^12,13,18^ The method can also be applied to functionalize other TMDs in order to enhance their properties for different applications.

## Experimental Methods

### Functionalization and Exfoliation of 2H-MoS_2_

**Figure 1:**
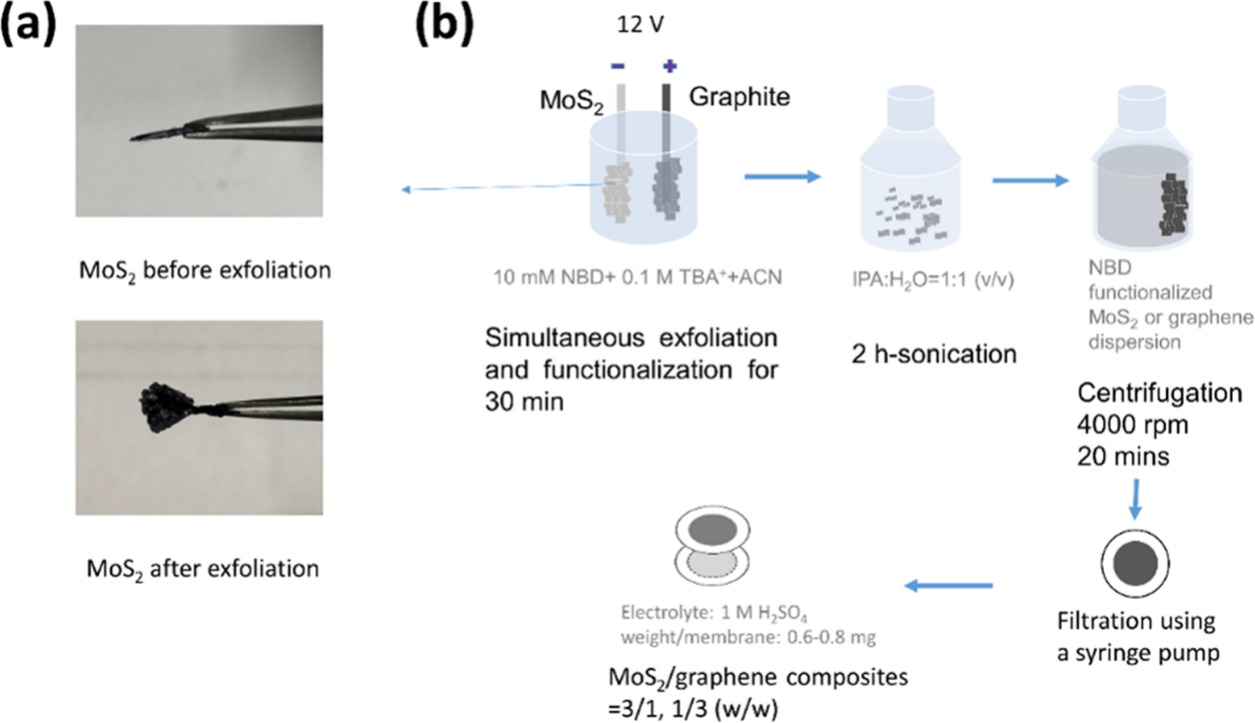
(a) Photographs of MoS_2_ crystals before and after exfoliation. (b) Schematic of the simultaneous exfoliation and functionalization procedure.

The electrochemical functionalization and exfoliation of 2H-MoS_2_ were performed using a natural MoS_2_ crystal as the cathode and graphite foil as the anode. The aryl diazonium salt used was 4-nitrobenzenediazonium tetrafluoroborate (NBD). Ten mM of NBD and 0.1 M of [TBA][PF_6_] (tetrabutylammonium hexafluorophosphate) were dissolved in acetonitrile, which was used as an electrolyte for the simultaneous electrochemical exfoliation and functionalization. The MoS_2_ crystal and the graphite foil were kept at a distance of 2 cm apart, and a voltage of 12 V was applied between them. After 30 min, the expanded fct-EEM and fct-EEG were dispersed separately in a mixture of isopropanol (IPA) and H_2_O (1:1 v/v) under the assistance of ultrasonication (2 h). The dispersion was then centrifuged at 4000 rpm. The supernatants of the dispersions were used to fabricate the composite electrodes. The unfunctionalized electrochemically exfoliated MoS_2_ (EEM) and graphene (EEG) were fabricated by using the same method without adding NBD to the solvent. The images of expanded MoS_2_ before and after the expansion as well as the production procedures can be seen in Figure 1.

### Fabrications of Composite Electrodes for Coin Cells

To produce supercapacitor electrodes, the fct-EEM and fct-EEG supernatants were mixed at gravimetric ratios of 1:3 and 3:1, respectively. The mixed dispersion was then ultrasonicated for 10 min to aid the mixing. The mixture was then filtered through poly(tetrafluoroethylene) (PTFE) membranes with diameters of 13 mm using a syringe pump. The loading was determined by weighing the PTFE membrane before and after the filtration. The weight of the active material on the composite electrode ranged from 0.6 to 0.8 mg. Two identical composite membranes were then stacked back to back with the PTFE sides facing each other, with the PTFE membranes serving as the separator. The stacked composite membranes were then sealed in a standard CR2032 coin cell after adding 1 M of H_2_SO_4_ electrolyte. This method has been demonstrated in previous papers.^19−21^ The pristine EEM/EEG composite electrodes and coin cells were fabricated by using the same method.

### Characterization Techniques

Raman spectroscopy was performed using a Renishaw inVia microscope with a 514 nm excitation laser with a power of 1 mW and a 100× objective and a grating of 2400 L/mm. For atomic force microscopy (AFM) imaging, a JPK NanoWizard atomic force microscope was operated in noncontact mode. Scanning electron microscopy (SEM) was carried out with an accelerating voltage of 10 kV. XPS was conducted using an ESCA2SR spectrometer (ScientaOmicron GmbH) equipped with a monochromated Al Kα radiation (1486.6 eV, 20 mA emission at 300 W, 1 mm spot size) with a base vacuum pressure of ∼1 × 10^–9^ mbar. A low-energy electron flood source (FS40A, PreVac) was used to achieve charge neutralization. C–C in the C 1s photoelectron peak at 285 eV was used to perform binding energy scale calibration. Analysis and curve fitting were performed using Voigt-approximation peaks using CasaXPS. The X-ray diffraction (XRD) patterns for MoS_2_ and graphene were acquired using the PANalytical X’Pert Pro diffractometer with Cu Kα radiation with a step size of 0.033° and a scan step size of 530 s/step.

### Electrochemical Measurements

Electrochemical measurements (CV, GCD, and EIS) on the composite coin cells were carried out using an Ivium CompactStat. CV scans were carried out at a scan rate ranging from 5 to 1000 mV s^–1^. GCD was conducted using a current density ranging from 0.5 to 10 A g^–1^. EIS was carried out at frequencies ranging from 0.01 Hz to 100 kHz with an amplitude of 5 mV.

## Results and Discussion

### Characterization

**Figure 2:**
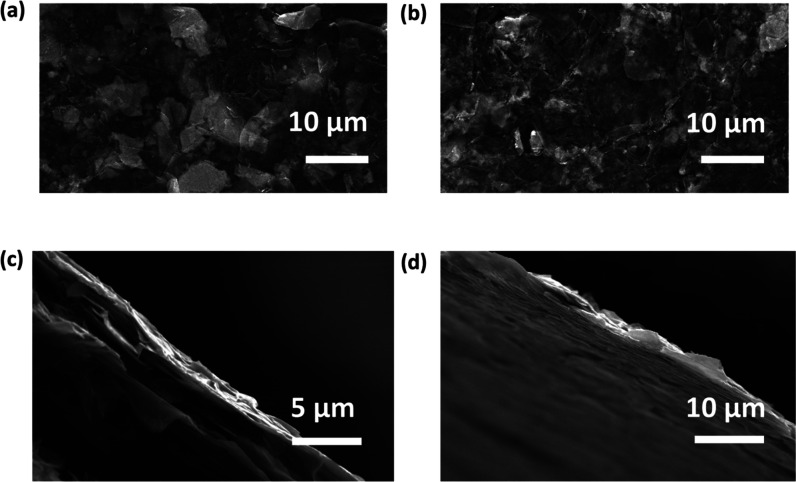
(a) SEM images of the surface of fct-EEM/fct-EEG and (b) EEM/EEG composite membrane. (c) and (d) Images are the SEM images of the cross sections of panels (a) and (b), respectively. The mass ratios of fct-EEM/fct-EEG and EEM/EEG are both 1:3.

The size and morphology of the fct-EEM and EEM flakes, as well as their composite membranes, were investigated using both SEM and AFM. The EEM/EEG composites (Figure 2a) show increased restacking compared to the fct-EEM/fct-EEG composites (Figure 2b), while the fct-EEM and fct-EEG composites have a looser structure, which is easier for the ions to get through the layers. As can be seen in Figure 2c,d, the fct-EEM/fct-EEG membrane is wavy and has layered structures, while the EEM/EEG composite membrane is more compact and flatter. This is because fct-EEG has more creases on the surface as a result of strain caused by intense functionalization. It is suggested that the wrinkled structures of fct-EEM/fct-EEG composites benefit the electrochemical performance of the composite electrodes by providing more edges and channels for the ions. The composite membranes had an average thickness of 2 μm, as measured by SEM.

**Figure 3:**
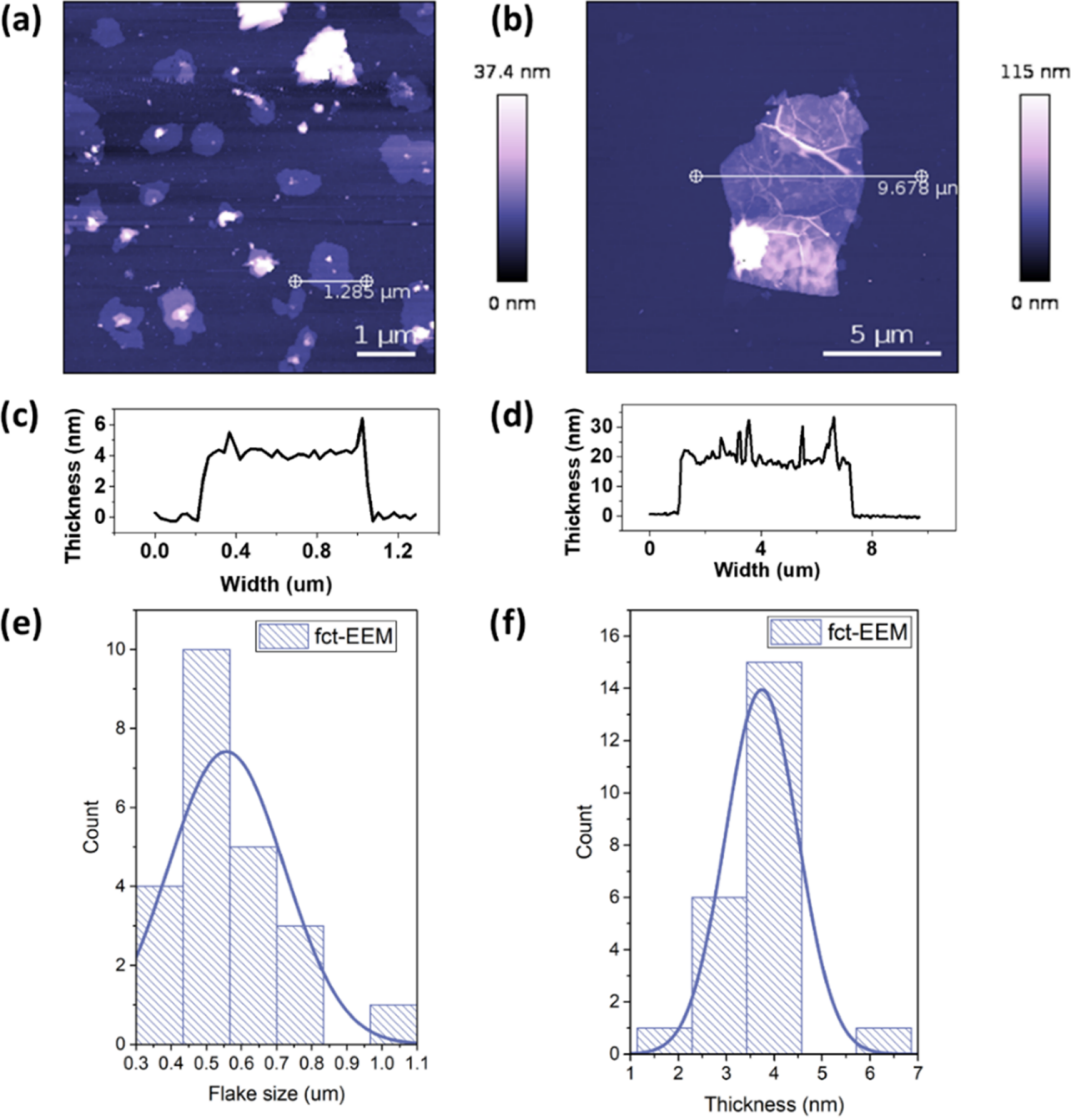
AFM images of (a) fct-EEM flakes and (b) fct-EEG flakes. Panels (c) and (d) are the height profiles measured along the lines in panels (a) and (b), respectively. Panels (e) and (f) are the distribution of the flake size and the thickness for fct-EEM, respectively.

Representative AFM images of the fct-EEM and fct-EEG are shown in Figure 3a,b, respectively, with height profiles for chosen flakes shown in Figure 3c,d. The thickness of fct-EEM (∼4 nm) is much thinner than that of EEM (∼60 nm, as shown in Figure S1a), which indicates that the aryl diazonium salts aided the exfoliation process, leading to a thinner flake. The diazonium salts also slightly aided the exfoliation of graphite; the thickness of graphite reduced from ∼500 nm (Figure S1b) to around 100 nm (Figure 3b). The distribution of the lateral flake size and the thickness of fct-EEM were obtained by measuring the flakes in the AFM images (Figure 3e,f). The thickness of the fct-EEM flakes is around 4 nm. Compared with unfunctionalized EEM (Figure S3), fct-EEM has smaller flake sizes (∼0.5 μm). The fct-EEG also has more flakes with small sizes than the EEG does (Figure S3). It is suggested that this is because the functionalization helps with the dispersion of the flakes in IPA and water and thus it is easier to obtain small flakes during the ultrasonication.

**Figure 4:**
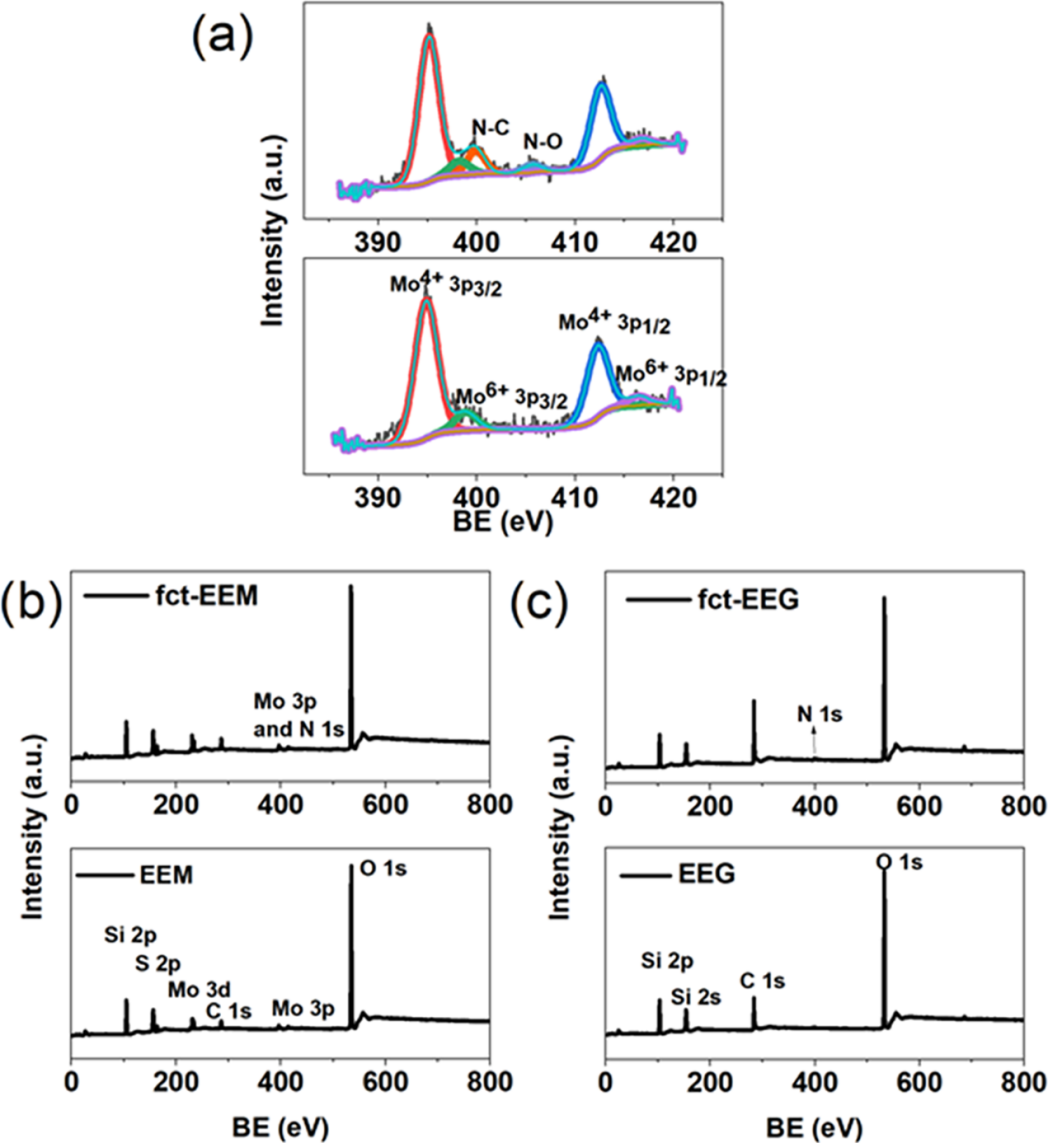
(a) High-resolution XPS spectra of the Mo 2p region for fct-EEM (top) and EEM (bottom). (b, c) Survey XPS spectra of indicated samples.

Figure 4a shows the Mo 3p core level from XPS of fct-EEM and EEM. Two extra peaks of N–C (399.92 eV) and N–O (405.62 eV) from the NBD are observed after the functionalization of 2H-MoS_2_ with NBD.^18^ This indicates that the chemically inert 2H-MoS_2_ can be functionalized electrochemically without any pretreatment (which can be damaging to the MoS_2_ surface or can change the band gap of MoS_2_). The graphene obtained by exfoliating graphite anodically was also functionalized. In Figure 4b, the XPS spectra of the N 1s region exhibit two characteristic peaks at 400.63 and 405.82 eV, which are also attributed to the N–C bond and N–O bond from the nitro groups of NBD. The presence of the N 1s peak of fct-EEM and fct-EEG in Figure 4c,d indicates the existence of the N from NBD.

**Figure 5:**
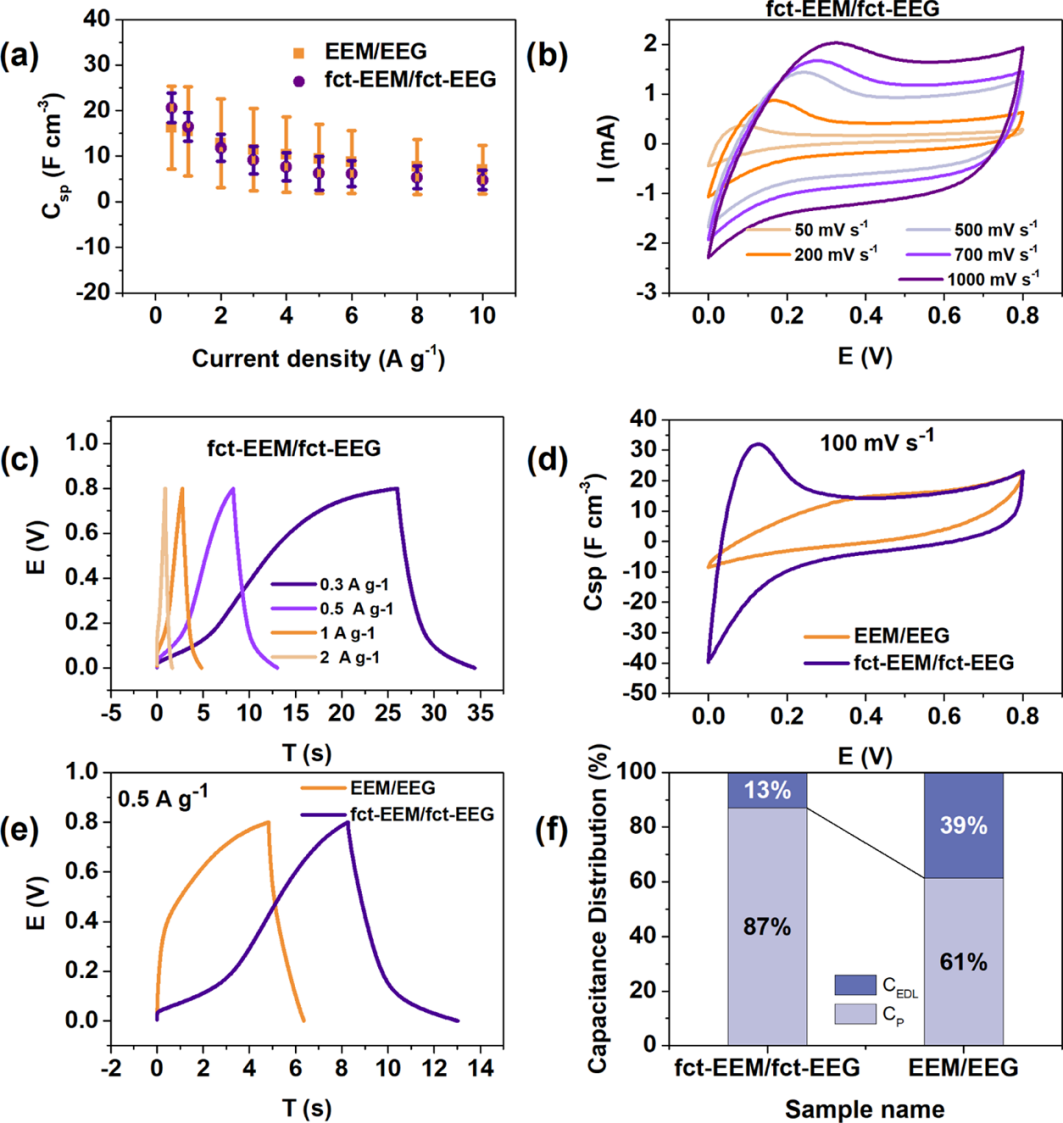
(a) Specific capacitances in F cm^–3^ of indicated samples versus current density. The values are the average specific capacitances of 3 devices that were obtained from GCD curves. The error bars were obtained from the standard deviations of the specific capacitances of 3 devices. (b) CV scans of fct-EEM/fct-EEG at different scan rates. (c) GCD curves of fct-EEM/fct-EEG at different current densities. (d) CV curves for indicated samples at a scan rate of 100 mV s^–1^. (e) GCD curves for indicated samples at a current density of 0.5 A g^–1^. (f) Capacitance distributions for fct-EEM/fct-EEG and EEM/EEG. The percentages were obtained by the ratio of the values in Table 2, which were calculated using the Trasatti methods. The Trasatti methods are shown in Figure S8. The mass ratios of MoS_2_ and graphene of the above composites are all 1:3.

As a nondestructive technique, Raman spectroscopy offers vast amounts of structural and electronic information. It has been widely used to study 2D materials, such as graphene and MoS_2_. The number of layers, the effects of strain, the presence of dopants and defects, and the crystallographic orientation can all be probed by Raman spectroscopy.^22^ The representative Raman spectra of EEM and fct-EEM are shown in Figure S4b. The two dominant peaks located at ∼380 cm^–1^ (^1^E_2g_) and ∼408 cm^–1^ (A_1g_) are attributed to the in-plane vibration and out-of-plane lattice expansion.^23^ The average peak position of ^1^E_2g_ is plotted against A_1g_ as shown in Figure 5a, and it was noted that both ^1^E_2g_ and A_1g_ are blue-shifted.

The blue shifts have occurred repeatedly for different batches of the same samples and were thus considered to be significant. The blue shift of ^1^E_2g_ indicates a reduction in the number of layers after functionalization, which is consistent with the AFM results.^24^ It also implies a compressive strain being generated due to the functionalization.^25^ The reduction in the number of layers is supposed to induce a red shift of the A_1g_ mode; however, here we observed a blue shift. Since a blue shift of A_1g_ also indicates a compressive strain,^25^ it is suggested that the effect from compressive strain has dominated so that we observe a blue shift as a result. The functionalization has also caused the D and G peak intensity ratios to increase as shown in Figure S6a,b, indicating an increase of defects that come from the functional groups. In addition, the error bars for fct-EEM are much smaller than those for EEM, which implies that the functionalization has increased the surface homogeneity of MoS_2_.

The XRD patterns of the MoS_2_ crystal, EEM, and fct-EEM are shown in Figure 5b. The peaks of EEM and fct-EEM are also broadened compared to those of the MoS_2_ crystal, indicating a rearrangement of the exfoliated MoS_2_ flakes. The XRD spectra before and after functionalization remain the same, which implies that the covalent attachment of NBD is not destructive and does not change the crystalline structure of MoS_2_. The functionalization has also increased the interlayer spacing of fct-EEG as shown in Figure S6c.

### Electrochemical Properties

To evaluate and compare the electrochemical properties of fct-EEM/fct-EEG and EEM/EEG composites, electrochemical tests including CV, GCD, and EIS were carried out. A plot of the specific capacitances for both fct-EEM/fct-EEG and EEM/EEG composites as a function of varying current densities is shown in Figure 5a. At 0.5 A g^–1^, the volumetric capacitance increases from 16.2 to 20.6 F cm^–3^ after functionalization. The volumetric capacitance of this fct-EEM/fct-EEG is comparable to that of previously reported MoS_2_ electrodes (Table 1). It can be seen that as the current density increases, the values of specific capacitances of both samples become closer. However, it is noticeable that the fct-EEM/fct-EEG is more reproducible in terms of specific capacitance (smaller error bars compared to that of EEM/EEG). A plausible explanation is that given enough time for functionalization, the surfaces of fct-EEM and fct-EEG are saturated with functional groups and thus each flake of EEM or EEG is uniform. CV and GCD measurements for fct-EEM/fct-EEG composites of other gravimetric ratios (3:1) were also performed (Figure S7), which show inferior electrochemical properties because of the decreased electric conductivity.

**Table 1:** Comparison of the Supercapacitor Performance of This Work with Other MoS2 Electrodes Reported Previously

electrode					
anode	cathode	electrolyte	current density/scan rate	volumetric capacitance (F cm^–3^)	references
MoS_2_/CNT	MnO_2_/CNT	1 M Na_2_SO_4_	10 mV s^–2^	6.5	(28)
MnO_2_/GNS	MoS_2_/GNS	PVA + Na_2_SO_4_	2 mV s^–1^	19.3	(29)
graphene/MoS_2_ composite		PVA + H_3_PO_4_	1.0 mA	19.44	(30)
MoS_2_/Ppy-2		KCl	0.5 A g^–1^	15.4	(31)
MoS_2_-rGO/MWCNT	rGO/MWCNT	PVA + H_2_SO_4_	0.5 A cm^–3^	4.8	(32)
fct-EEM/fct-EEG		H_2_SO_4_	0.5 A g^–1^	20.6	this work

Figure 5b shows CV curves of fct-EEM/fct-EEG at scan rates ranging from 50 to 1000 mV s^–1^. When at a lower scan rate (<200 mV s^–1^), a redox peak at ∼0.1 V is observed and the CV curves are not symmetric, indicating that the reactions are not reversible. The redox peak fades as the scan rate increases, and shapes of the CV curves exhibit quasi-rectangular shapes, which might be due to the fact that there is not sufficient time for the fct-EEM/fct-EEG to react at high scan rates, and thus the EDL capacitance dominates. The area of the enclosed CV curve increases as the scan rate increases, suggesting good capacitive behavior of fct-EEM/fct-EEG.^26^

Figure 5c displays the GCD curves of fct-EEM/fct-EEG at different current densities. The GCD curves deviated from ideal triangular shapes, which results from the redox reactions. The CV curves of fct-EEM/fct-EEG and EEM/EEG are compared in Figure 5d. There is no obvious redox peak observed for the CV curve of EEM/EEG, while there is one at ∼0.1 V for fct-EEM/fct-EEG. The redox peak for fct-EEM/fct-EEG indicates that the capacitance of fct-EEM/fct-EEG consists of both pseudocapacitance and EDL capacitance. In addition, the larger enclosed area of the CV curve for fct-EEM/fct-EEG compared to that for EEM/EEG implies a larger specific capacitance for fct-EEM/fct-EEG, which is consistent with the GCD curves shown in Figure 5e where fct-EEM/fct-EEG exhibits a larger time span.

To estimate the effects of functionalization on the capacitance contribution of the electrode, the Trasatti method was used to calculate the total specific capacitance (*C*_T_), electric double layer capacitance (*C*_EDL_), and the pseudocapacitance (*C*_P_).^27^ The results of the estimated specific capacitances are displayed in Table 2, and the percentages of capacitance distribution are shown in Figure 5f. The contribution of pseudocapacitance increases from 61 to 87% because of the redox reactions after functionalization. This result agrees with the redox peak of the CV curve of fct-EEM/fct-EEG shown in Figure 5d. It can be noticed that the estimated specific capacitances (Table 2) from the Trasatti methods are much larger than the tested specific capacitances shown in Figure 5a. This is because the specific capacitances from the Trasatti methods were calculated by fitting a straight line at a low scan rate. Theoretically, as the scan rate is close enough to 0 mV s^–1^, there is enough time for the ions to diffuse into the entire electrode, which is why the specific capacitance is not limited by the diffusion rate and has a larger specific capacitance than calculated.

**Table 2:** Table of *C*_T_, *C*_EDL_, and *C*_P_ in F cm^–3^ Obtained Using the Trasatti Method for the Indicated Samples

	*C*_T_ (F cm^–3^)	*C*_EDL_ (F cm^–3^)	*C*_P_ (F cm^–3^)
fct-EEM/fct-EEG	61.01	7.88	53.14
EEM/EEG	29.61	11.42	18.19

**Figure 6:**
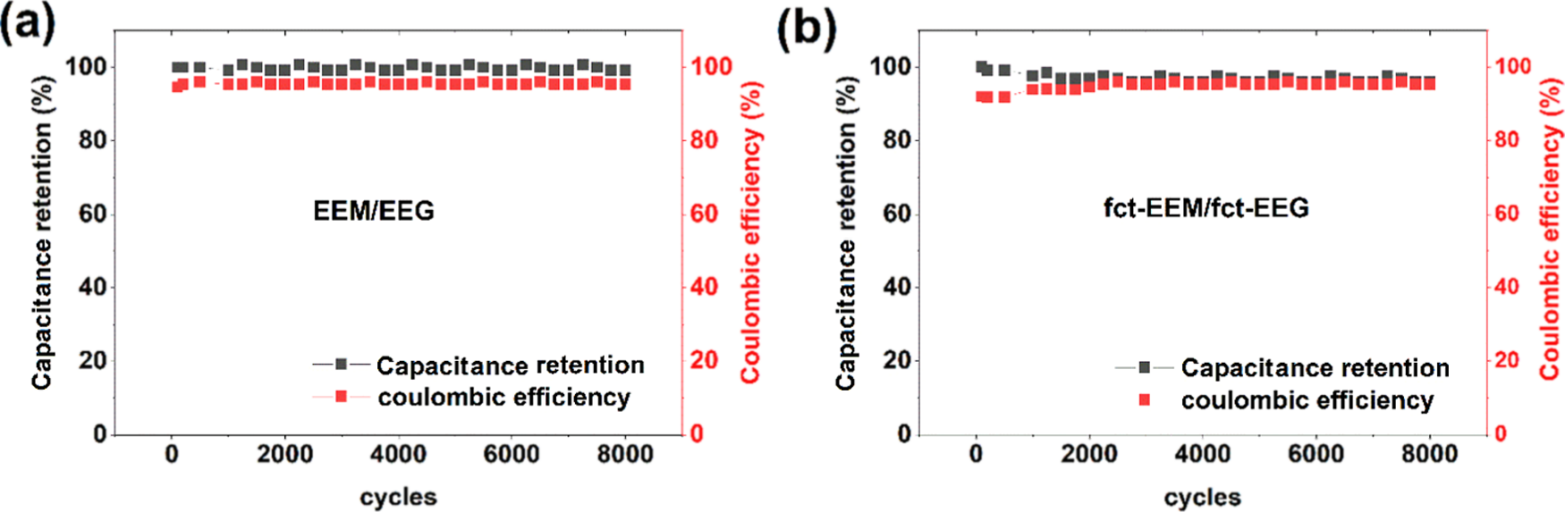
Capacitance retention and Coulombic efficiency of (a) EEM/EEG and (b) fct-EEM/fct-EEG, both of which were obtained by carrying out a GCD test at a current density of 2 A g^–1^.

The Coulombic stability and capacitance retention are used to evaluate the cyclic stability of the electrode materials. The capacitance retentions and the Coulombic efficiencies for fct-EEM/fct-EEG as well as EEM/EEG are shown in Figure 6a,b. The capacitance retentions for fct-EEM/fct-EEG and EEM/EEG are ∼96 and ∼99%, and the Coulombic efficiencies for them are both ∼95% after 8000 cycles. Both fct-EEM/fct-EEG and EEM/EEG display excellent capacitance retention and Coulombic efficiencies, which implies good stability for both materials. As expected, the capacitance retention for fct-EEM/fct-EEG is slightly lower than that for EEM/EEG because of the irreversible redox reactions in fct-EEM/fct-EEG, while EEM/EEG is closer to EDLC in terms of electrochemical properties.

**Figure 7:**
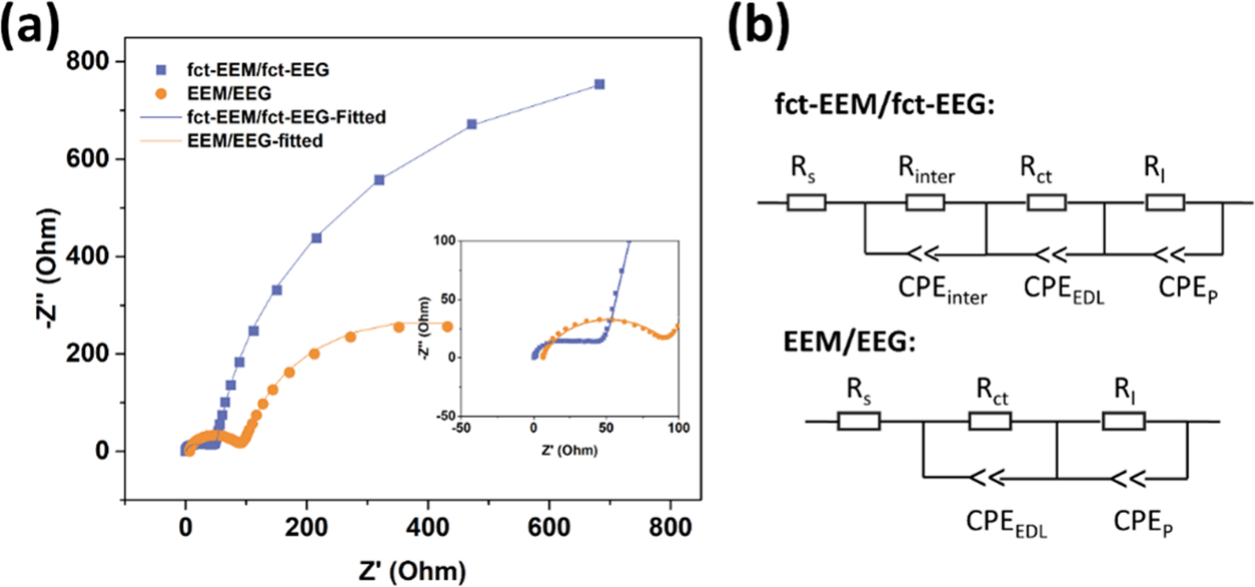
(a) Nyquist plots for fct-EEM/fct-EEG and EEM/EEG and (b) equivalent electric circuit for fct-EEM/fct-EEG and EEM/EEG.

Electrochemical impedance spectroscopy was carried out for fct-EEM/fct-EEG and EEM/EEG symmetric coin cells to study the charge transfer at the electrode/electrolyte interface. The Nyquist plots for both devices are shown in Figure 7a, and the equivalent electric circuits that were used to fit the Nyquist plots are shown in Figure 7b. *R*_s_ is ascribed to the equivalent series resistance (ESR). *R*_ct_ and CPE_EDL_ correspond to the charge-transfer resistance and the double layer resistance, while *R*_l_ and CPE_P_ refer to the leakage resistance and the pseudocapacitance, respectively.^33^ fct-EEEM/fct-EEG has a smaller ESR, which is 0.05 Ω compared to that of EEM/EEG (6.3 Ω). As evident in Figure 7a, EEM/EEG has a larger semicircle at the high-frequency region, and thus the charge-transfer resistance (*R*_ct_) for EEM/EEG (86.3 Ω) is larger than that of fct-EEM/fct-EEG (40 Ω). This might be due to the better wettability of fct-EEM/fct-EEG after the functionalization. Leakage resistance (*R*_l_) refers to the electrical resistance that limits or controls the flow of current through an unintended or undesired path in a circuit, which is usually quite large and thus can be neglected (614.4 Ω for EEM/EEG and 1628 Ω for fct-EEM/fct-EEG).^33^ There is an extra semicircle for the fct-EEM/fct-EEG at the high-frequency region, which can be attributed to the resistance (*R*_inter_) and the capacitance (CPE_inter_) for the interlayers.^34^ It is suggested that the presence of *R*_inter_ and CPE_inter_ is because fct-EEM and fct-EEG have smaller flakes, which means there are more paths and channels for the electrolytes to get through the electrode and form capacitance between the layers. In addition, the loose, layer-by-layer structures of fct-EEM/fct-EEG shown in Figure 2c also help with the formation of the interlayer capacitance, while EEM/EEG is more compact, and the layers are in better contacts with each other, and thus, the EEM/EEG composite can be regarded as a whole.

## Conclusions

In summary, we have demonstrated a convenient and efficient way of simultaneously electrochemically exfoliating and functionalizing 2H-MoS_2_ without any pretreatment and applied this material for usage in supercapacitor electrodes. The N–C bonds and the N–O bonds from the NBD were observed by XPS, implying the successful covalent functionalization of 2H-MoS_2_. Graphene was also functionalized simultaneously as the –NO_2_ bonds occurred after functionalization, and the presence of this material helped improve the electrical conductivity of the resulting electrodes. The average thickness of fct-EEM is around 4 nm, which is much thinner than that of the pristine EEM. This is attributed to the cations of NBD being intercalated into the layers of the MoS_2_ crystals and assisting with the exfoliation. We also performed electrochemical experiments (CV, GCD, and EIS) to investigate how the functionalization affects MoS_2_ and demonstrated an improvement in the energy storage capacity of this material. These devices are more reproducible after functionalization. This technique for direct 2H-MoS_2_ functionalization reported for the first time has shown evidence of producing high-quality and thin MoS_2_ flakes. It has the potential to functionalize other TMDs that are also difficult to functionalize because of their chemical inertness. The technique also allows for the aryl diazonium salts to be easily replaced by other types of functional groups in order to adapt to different applications accordingly.

## Supporting Information Available

AFM images of EEM and EEG; histograms of flake size distributions for EEM, EEG, and fct-EEG; representative Raman spectrum for fct-EEG and EEG; histograms of intensity ratios of D and G peaks for EEG and fct-EEG; XRD patterns for graphite, EEG, and fct-EEG; ^1^E_2g_ peak position versus A_1g_ peak position for fct-EEM and EEM; representative Raman spectrum for fct-EEM and EEM; CV scans for fct-EEM/fct-EEG composites of a weight ratio of 3:1; specific capacitances of fct-EEM/fct-EEG of a weight ratio of 1:3 versus current densities; Trasatti method for the calculation of capacitance contributions; SEM images of MoS_2_ crystals before and after exfoliations; and schematics of the production procedures (PDF)
