# Supplementary material for: Simultaneous Electrochemical Exfoliation and Functionalization of 2H-MoS2 for Supercapacitor Electrodes
Source: ACS Appl Nano Mater. 2023 Oct 2;6(19):18062–70. doi: 10.1021/acsanm.3c03322 (PMC10580280; doi:10.1021/acsanm.3c03322)
Supplement: Supplementary file 1 — an3c03322_si_001.pdf [file an3c03322_si_001.pdf]

Supporting Information

Simultaneous Electrochemical Exfoliation and Functionalization of 2H-MoS_2 for Supercapacitor Electrodes

Yuling Zhuo, Ian A. Kinloch and Mark A. Bissett*

Department of Materials, National Graphene Institute, University of Manchester, Oxford Road, Manchester, M139PL, United Kingdom

E-mail: Mark.Bissett@Manchester.ac.uk

AFM Analysis

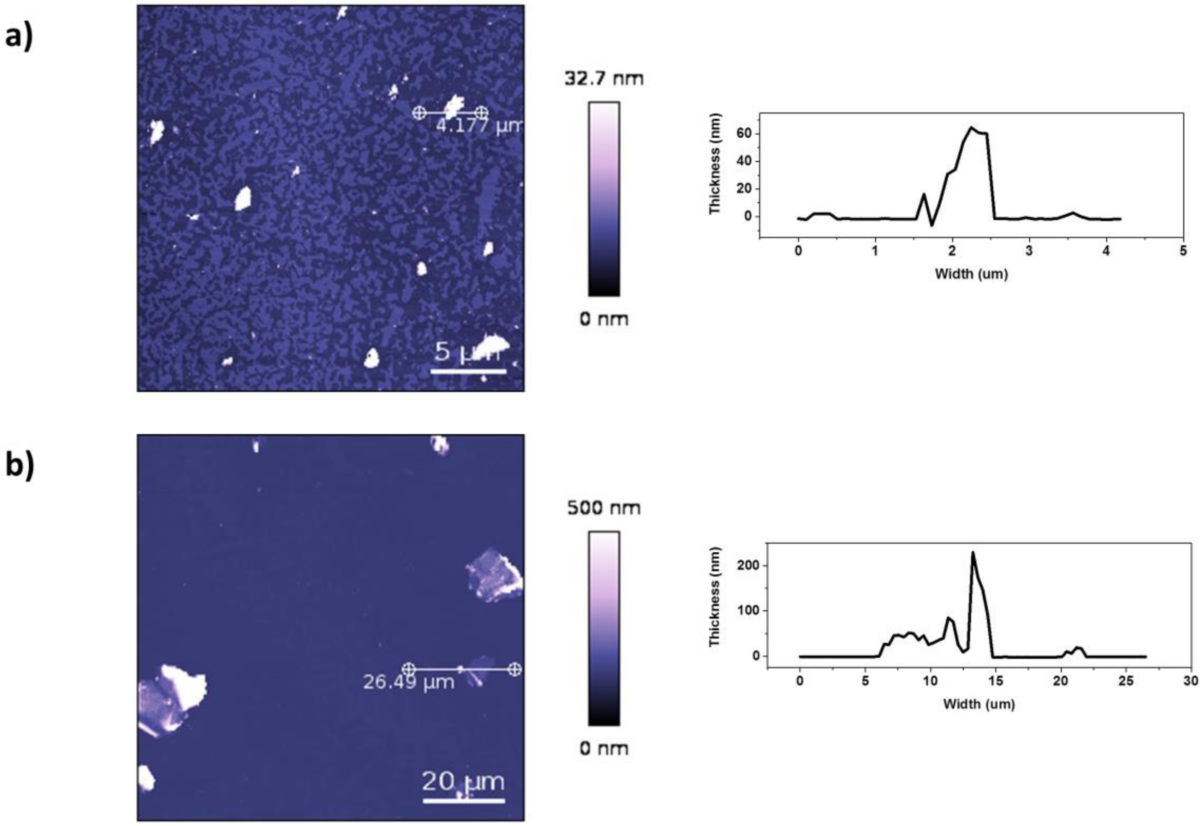

Figure S1: AFM images of a) electrochemically exfoliated MoS_2 (EEM) and b) electrochemically exfoliated graphene (EEG).

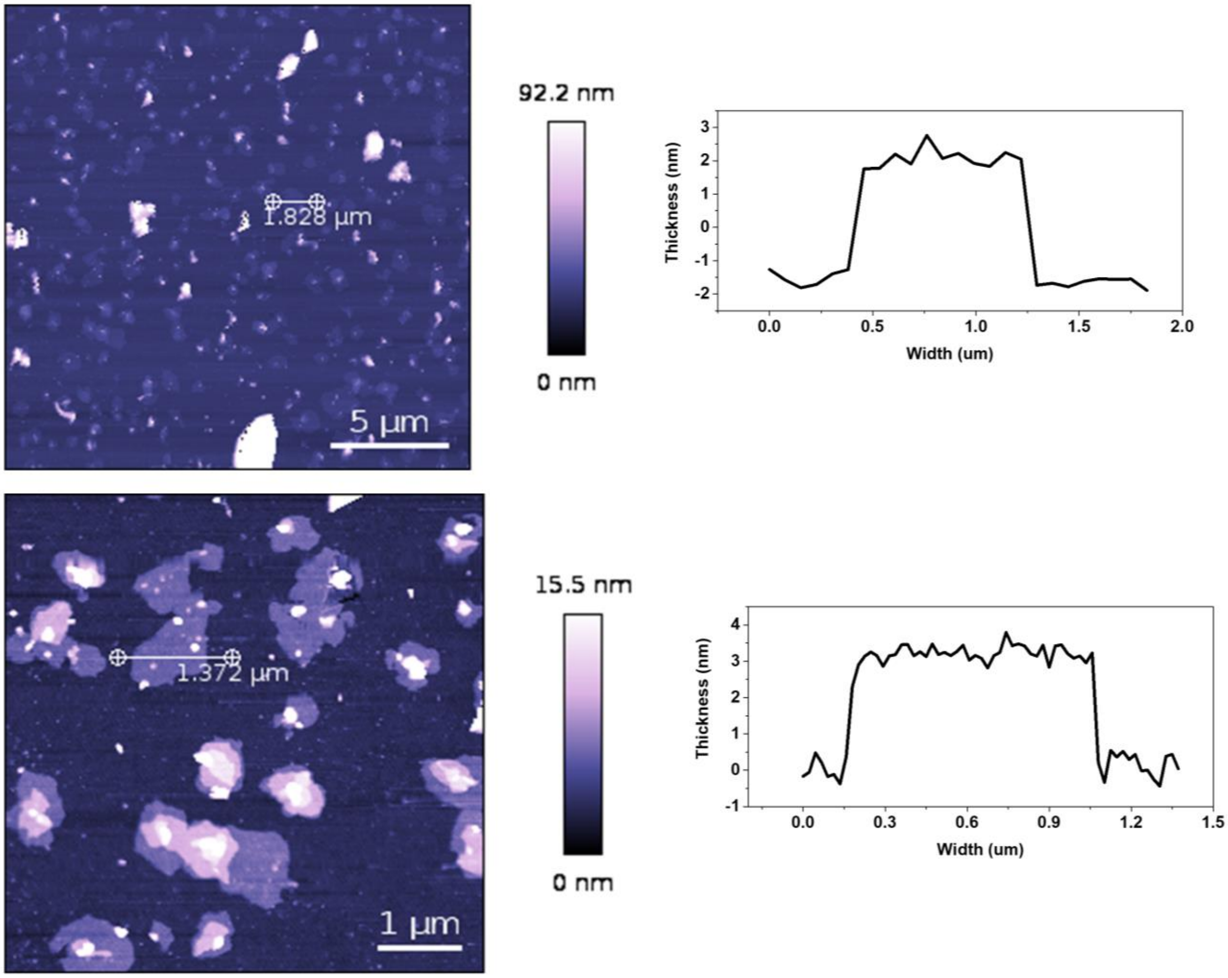

Figure S2: Additional AFM images of fct-EEM along with height profiles.

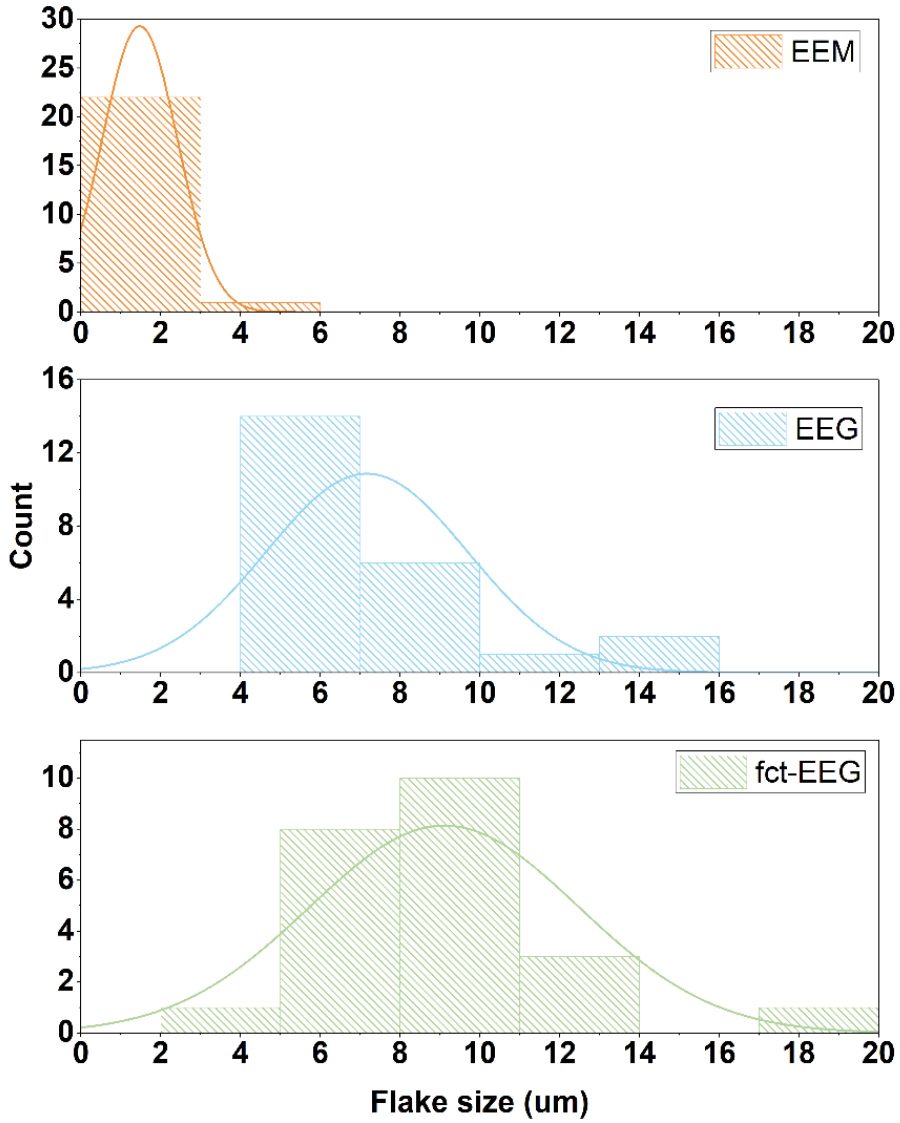

Figure S3: Flake size distributions for EEM, EEG and fct-EEG.

Raman and XRD Analysis

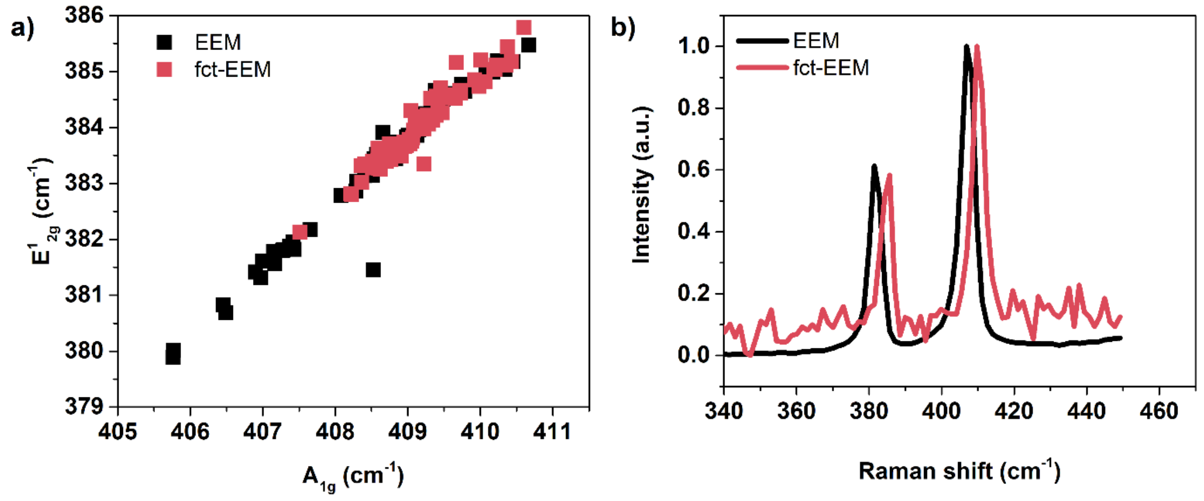

Figure S4: a) E^1_{2g} peak position versus A_{1g} peak position for fct-EEM and EEM. b) Representative Raman spectrum for fct-EEM and EEM.

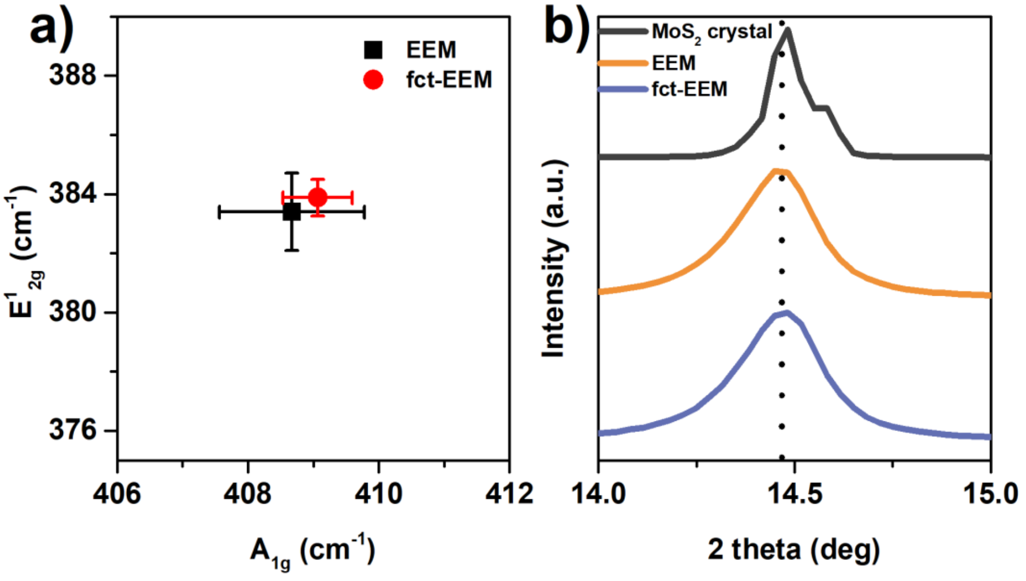

Figure S5: a) The average position of E^1_{2g} mode against that of the A_{1g} mode. The position of E^1_{2g} with respect to that A_{1g} from each Raman spectrum in the Raman mapping are also plotted, which are shown in Figure S5a. b) XRD pattern for indicated samples.

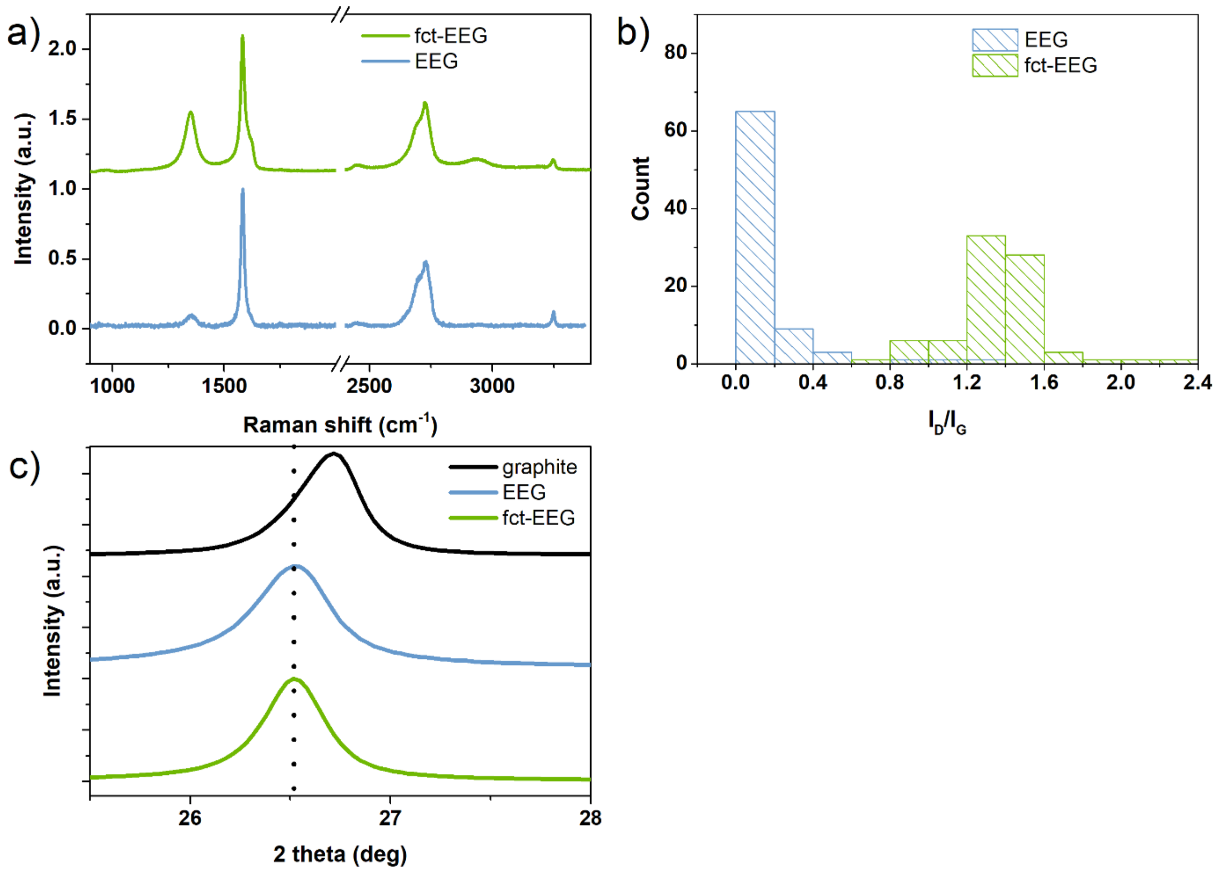

Figure S6: a) Representative Raman spectrum for fct-EEG and EEG. b) Histograms of intensity ratios of D and G peak for EEG and fct-EEG. c) XRD patterns for graphite, EEG and fct-EEG.

Capacitance Contribution

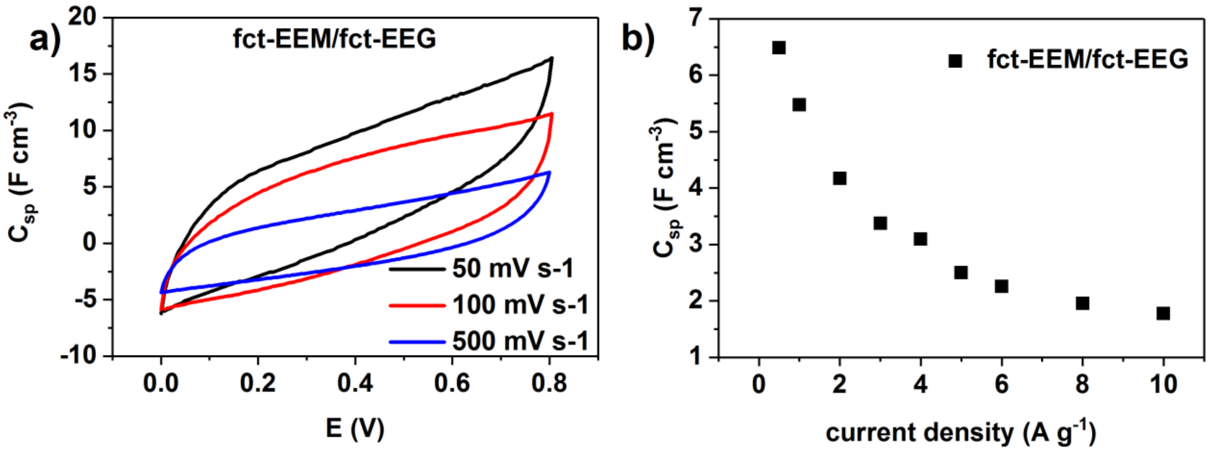

Figure S7: a) Cyclic voltammetry for fct-EEM/fct-EEG composites of a weight ratio of 3:1. b) Specific capacitances of fct-EEM/fct-EEG of a weight ratio of 1:3 versus current densities.

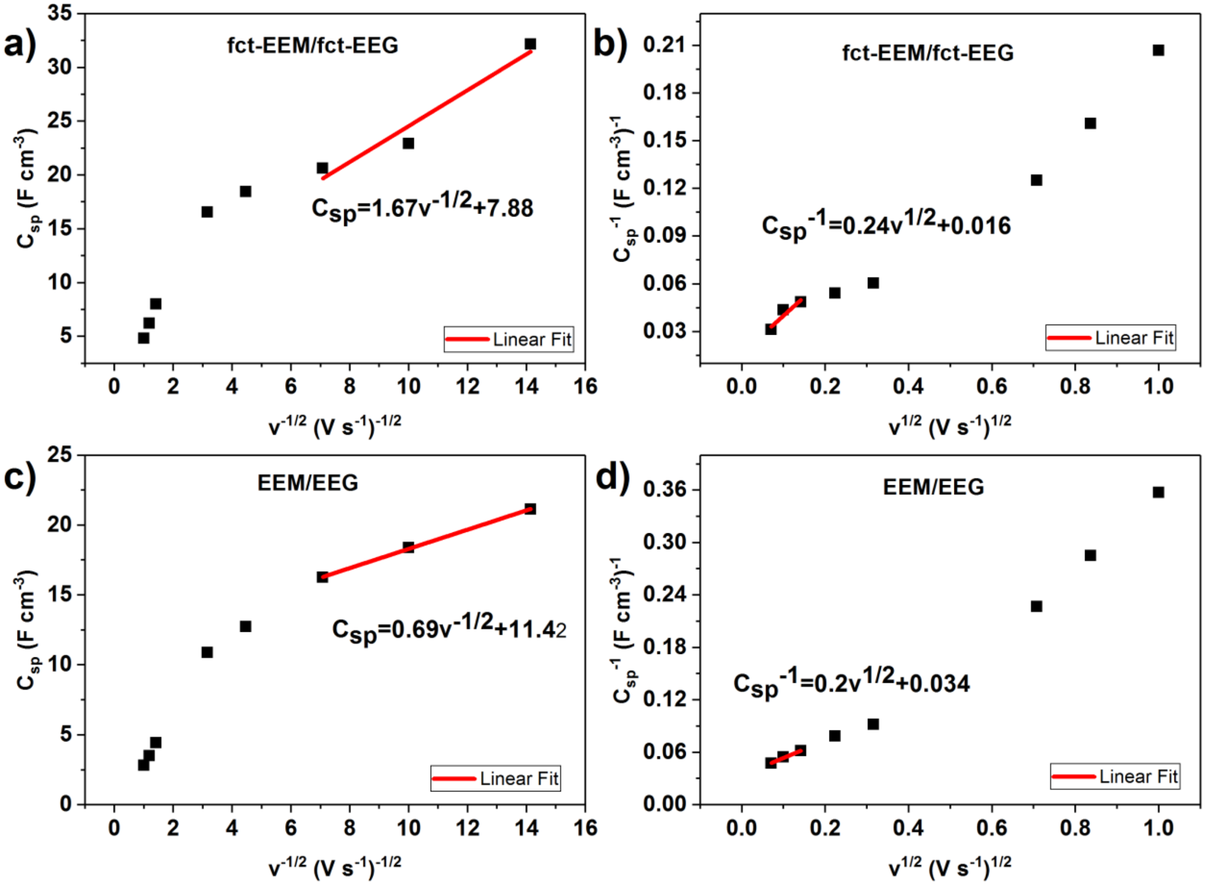

Figure S8: Trasatti method to calculate contribution to capacitance values. a) and c) C_{sp} as a function of $v^{-1/2}$ for fct-EEM/fct-EEG and EEM/EEG. b) and d) C_{sp}^{-1} as a function of $v^{1/2}$ for fct-EEM/fct-EEG and EEM/EEG. The red lines are the linear fitting of the curve at low scan rates (5 mV s^{-1} to 20 mV s^{-1}).

The percentage of capacitance contributions from electric double layer capacitance (EDLC) and pseudocapacitance (P) can be calculated using the following equations:

$$C^{-1} = k_1 \cdot v^{1/2} + C_T^{-1}$$

where C is the specific capacitance in F cm^{-3} which is calculated from enclosed area of the CV curves, k_1 a constant, v the scan rate (V s^{-1}) and C_T the total capacitance (F cm^{-3}).

$$C = k_2 \cdot v^{-1/2} + C_{EDL}$$

where k_2 is a constant and C_{EDL} the electric double layer capacitance (F cm^{-3}). By first plotting C^{-1} against $v^{1/2}$ and C against $v^{-1/2}$ followed by linear fittings at low scan rates, C_T and C_{EDL} can be obtained using the above equations. The difference between C_T and C_{EDL} gives the maximum pseudocapacitance (C_P).
